# HeadSLAM: Pedestrian SLAM with Head-Mounted Sensors

**DOI:** 10.3390/s22041593

**Published:** 2022-02-18

**Authors:** Xinyu Hou, Jeroen Bergmann

**Affiliations:** Natural Interaction Lab, Department of Engineering Science, University of Oxford, Oxford OX1 3PJ, UK; xinyu.hou@eng.ox.ac.uk

**Keywords:** simultaneous localisation and mapping (SLAM), wearable sensors, FootSLAM, pedestrian navigation, inertial measurement unit

## Abstract

Research focused on human position tracking with wearable sensors has been developing rapidly in recent years, and it has shown great potential for application within healthcare, smart homes, sports, and emergency services. Pedestrian Dead Reckoning (PDR) with Inertial Measurement Units (IMUs) is one of the most promising solutions within this domain, as it does not rely on any additional infrastructure, whilst also being suitable for use in a diverse set of scenarios. However, PDR is only accurate for a limited period of time before unbounded errors, due to drift, affect the position estimate. Error correction can be difficult as there is often a lack of efficient methods for calibration. HeadSLAM, a method specifically designed for head-mounted IMUs, is proposed to improve the accuracy during longer tracking times (10 min). Research participants (*n* = 7) were asked to walk in both indoor and outdoor environments wearing head-mounted sensors, and the obtained HeadSLAM accuracy was subsequently compared to that of the PDR method. A significant difference (*p* < 0.001) in the average root-mean-squared error and absolute error was found between the two methods. HeadSLAM had a consist lower error across all scenarios and subjects in a 20 h walking dataset. The findings of this study show how the HeadSLAM algorithm can provide a more accurate long-term location service for head-mounted, low-cost sensors. The improved performance can support inexpensive applications for infrastructureless navigation.

## 1. Introduction

In recent years, human position tracking technology has drastically changed modern life by offering location information for a variety of scenarios. The field is still developing at an incredible pace, with new insights continuously being reported. These Location-Based Services (LBS) are able to provide accurate location tracking of people, and they have started to be adopted in a range of different applications, such as smart environments, healthcare [1], pedestrian navigation, and emergency services [2]. It has also facilitated new ways of human–environment interaction, in which positional information could be leveraged to create responsive systems. For example, smart homes can use information from LBS to make better decisions on how to support those living in these spaces [3]. These responsive interactions might be particularly interesting for healthcare settings, such as care homes. For example, services can be deployed to provide assistance to the elderly by identifying their daily routines and establishing care plans that are specifically developed around the patient, instead of the other way around. This could allow for more personalised healthcare, by the integration of the mobility patterns of users and mapping of their physical behavioural routines.

Another area of application for LBS is for those who are interested in monitoring or optimising their physical performance during sports. Physical activity through sports participation has become an essential part of ensuring healthy living in today’s world, and key to this is the tracking of workload [4]. LBS provide a suitable method to determine the external loading during sports activities. This can then subsequently be combined with objective measurements of internal loading to accurately provide a complete picture of an athlete’s workload [5].

The technology used to offer LBS needs to take into account the constraints that are set by the application. The Global Navigation Satellite System (GNSS) is widely used in outdoor position tracking solutions. GNSS allows small electronic receivers to determine their locations with high precision using time data transmitted along a line of sight by radio signals from satellites. However, due to signal blockage or strong multipath propagation, satellite signals such as the Global Positioning System (GPS) are often unavailable or degraded in critical environments, which include indoor, underground, or urban canyons [6]. To overcome the challenge of pedestrian position tracking in indoor environments, several methods have been developed by researchers, which could be classified into building-dependent and building-independent methods. Building-dependent methods include techniques that require access to the building’s infrastructures such as WiFi [7], cellular, or Bluetooth, as well as approaches requiring dedicated infrastructures, such as Radio Frequency Identification (RFID) [8], Ultra-wideband (UWB) [9,10], infrared, radar [11,12], ZigBee, Visible Light Communication (VLC), and acoustic signals. For example, Reference [13] utilised multi-source information from markers, optical flow, ultrasound, and an inertial sensor to provide a tracking solution. Unfortunately, most of them thus rely on external aiding signals, information, or infrastructures, which makes it impossible to leverage them in places where these signals are severely affected or where there is no specific infrastructure available.

Considering the previous mentioned difficulties, building-independent self-contained position tracking methods, such as Inertial Measurement Units (IMUs), become a more suitable choice. IMUs are small, discreet, portable, energy efficient, and easy to implement in everyday objects and can be acquired at a low cost. Many IMU solutions apply Pedestrian Dead Reckoning (PDR) methods, which utilise the features of human locomotion to re-calibrate the data [14,15]. For example, Foxlin leveraged the foot’s rest phase to inject Zero-velocity Updates (ZUPTs), as pseudo measurements, into a filter to reduce the estimation error [16]. However, these techniques are only accurate across a “short” time period, and they become prone to drifting errors when the measurement time increases. The drift error and the lack of a reliable calibration reference subsequently limits the utility of this method. Thus, it is essential to find a pedestrian tracking method that has a lower error when tracking is applied over longer periods of time (e.g., 10 min).

Simultaneous Localisation And Mapping (SLAM) is a computational problem of constructing or updating a map of an unknown environment, while tracking the location of the object at the same time [17]. Current SLAM methods can generate accurate location tracking trajectories and environment maps by using reference landmarks to reduce errors. These landmarks can be observed by exteroceptive sensors, such as cameras [18], laser range finders [19], LiDAR [20], or sonar [21]. However, these extra sensors also come at an added cost, and image-capturing devices face the problem of privacy or security threats when used in a private environment. Michael Angermann and Patrick Robertson previously proposed the FootSLAM algorithm, which only uses accelerometers and gyroscopes embedded in a foot-mounted IMU. FootSLAM applies a Rao-Blackwellised particle filter to build a probabilistic transition map. This approach was able to prevent unbounded error growth, and they presented two subsequent extensions of this (PlaceSLAM and FeetSLAM) [22]. Susanna Kaiser and Estefania Munoz Diaz subsequently created PocketSLAM, which is a combination of a pocket navigation system with a FootSLAM method [23]. They showed that it was possible to reduce drift with this technique.

The SLAM methods provide an opportunity to leverage small IMUs to create useful location data. This kind of hardware can easily be embedded into clothing or everyday objects. A survey that asked potential users how a wearable device should look showed that wearable sensor technologies were expected to be small, discreet, unobtrusive, and preferably incorporated into everyday objects [24], which indicates that the acceptability for an embedded positional tracking system could be rather high.

The selection of the location where this sensor system can be placed should take into account the acceptability of the sensor placement. The head provides an interesting location to attach sensors to, as there are several worn objects that could easily be adapted for monitoring purposes. It also provides an opportunity for the integration of sensors in everyday objects that would be acceptable for use in healthcare, sports, or work. Potential objects that the sensor system could be integrated into are glasses, mouth guards, face masks, helmets, earrings, earphones, hearing aids, or even caps.

In previous work, we proposed a PDR method especially for head-mounted IMUs (HeadPDR) [25], which could generate accurate results for short trajectories, across brief recording periods (<1 min). In this paper, SLAM was combined with the previously proposed HeadPDR method to determine if this would yield lower position errors compared to the application of the PDR method on its own. The aim was to test this for both indoor and outdoor environments, which simulate a sports and healthcare setting. The contribution of the work consists of proposing a SLAM method for head-mounted sensors, which only requires low-cost portable IMU data and remains accurate over longer time periods. The rest of the paper is organised as follows: Section 2 describes the PDR and HeadSLAM methods. Section 3 outlines the experimental conditions. The results are shown and analysed in Section 4, whilst Section 5 and Section 6 conclude the research and discuss the future applications.

## 2. Methods

**Figure 1:**
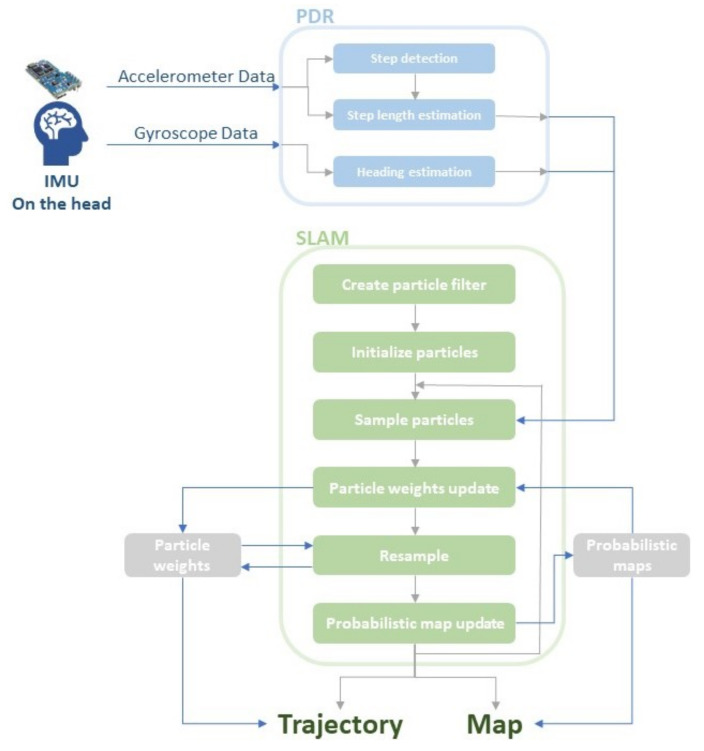
Overview of the HeadSLAM method. The Simultaneous Localisation And Mapping (SLAM) step is performed after the Pedestrian Dead Reckoning (PDR) in order to create a map based on the data collected from head-mounted inertial measurement units. The grey arrows show the sequence of the flowchart. The dark blue arrows represents the data or the variables’ usage or update.

The HeadSLAM method consists of two distinct stages: (i) PDR and (ii) SLAM. The PDR estimates the heading direction and the length of each step based on raw accelerometer and gyroscope data. This is then fed into SLAM to generate a suitable map. The whole process is shown in Figure 1.

### 2.1. PDR

A PDR algorithm designed for head-mounted sensors was proposed in [25] and was adopted in this study. It uses Step-and-Heading Systems (SHSs). The SHSs output a series of step vectors by detecting each step of the user, estimating the length and direction of it. This information is then integrated across each step to obtain a complete trajectory. The next position of the pedestrian could then be estimated with (1). The current position after the $k_{th}$ step $\left(x_{k},y_{k}\right)$ is input into the equation. $l_{k+1}$ and $\varphi_{k+1}$ represent the step length in meters (*m*) and the forward direction of the next step given in degrees (${}^{\circ }$). The iterative process provides an updated estimate of the *x* and *y* positions, which reflect the 2D space in which the person is moving.

(1) $$\begin{array}{c} x_{k+1}=x_{k}+l_{k+1}\times \sin\left(\varphi_{k+1}\right)\\ y_{k+1}=y_{k}+l_{k+1}\times \cos\left(\varphi_{k+1}\right) \end{array}$$

#### 2.1.1. Step Detection

The step detection is updated in a comparable manner to the method used in [25], which also adopted a peak detection approach to identify a step at a heel strike. The vertical acceleration (*z*-axis) is first filtered by applying a first-order Low-Pass Filter (LPF) with a cut-off frequency set to 2 Hz to eliminate any high-frequency noise. This is sufficient to capture the general walking motion of most users. Peaks and valleys in the filtered signal are then identified. If the value difference between a peak and its subsequent valley exceeds the predetermined threshold (0.5 m/s${}^{2}$), the time interval between the previous and subsequent valley will be recognised as a step.

#### 2.1.2. Step Length Estimation

Weinberg’s model [26] was used as the step estimator:

(2) $$step\_length=k\cdot \sqrt[{4}]{a_{max}-a_{min}}$$

where *k* is a constant coefficient for unit conversion, whilst $a_{max}$ and $a_{min}$ (ms${}^{-2}$) indicate the maximum and minimum acceleration measured in the z-direction for a single step. It was proven to perform best for personalised sets of constants compared to 12 representative step length estimation models [27].

#### 2.1.3. Heading Estimation

A quaternion-based derivation of the explicit complementary filter proposed by Mahony et al. [28] was adopted as the heading estimator, which considers the problem of obtaining good attitude estimates from measurements obtained from typical low-cost inertial measurement units.

The Mahony algorithm can also fuse the magnetometer data into the calculation. However, this study did not include any magnetometer data in the final estimation, because of the unpredictable magnetic interference in indoor spaces, the noise in these low-cost sensors, and the expectation for a longer battery life of the wearable devices [29,30,31].

### 2.2. HeadSLAM

The length and heading direction of each step (estimated by the PDR) are fed into the SLAM for re-calibration. The output from this is subsequently used to build the map.

In the SLAM algorithm, the two-dimensional space is first divided into a grid of adjacent hexagons of a given radius. The Rao-Blackwellised Particle Filter (RBPF), which is applied in the FastSLAM algorithm [32], is then used. The SLAM problem was decomposed into a pedestrian localisation problem and a mapping problem conditioned on the pedestrian’s position (pose). The posterior can be simplified as:

(3) $$p\left(P_{0:k},M|Z_{1:k}\right)=p\left(M|P_{0:k}\right)\cdot p\left(P_{0:k},Z_{1:k}\right)$$

where **P** and **M** represent the pose and the map and $Z_{k}$ is a noisy measurement of the difference between $P_{k-1}$ and $P_{k}$, which is the step vector estimated from the previous PDR layer. The pose could be estimated recursively:

(4) $$p\left(P_{0:k}|Z_{1:k}\right)\propto p\left(Z_{k}|P_{k-1:k}\right)\cdot p\left(P_{k}|P_{0:k-1}\right)\cdot p\left(P_{0:k-1}|Z_{1:k-1}\right)$$

$p(Z_{k}|P_{k-1:k})$ is the likelihood function, which adopts a normal distribution to draw possible poses after each step. The pose transition function $p(P_{k}|P_{0:k-1})$ is computed by marginalizing over the map. Integrating it yields:

(5) $$I^{i}\propto \frac{N_{\tilde{h}}^{\tilde{e}}+\alpha_{\tilde{h}}^{\tilde{e}}}{N_{\tilde{h}}+\alpha_{\tilde{h}}}$$

where $N_{\tilde{h}}^{\tilde{e}}$ is the number of times the *i*-th particle crossed edge $\tilde{e}$ and $N_{\tilde{h}}$ is the sum of the crossed times of all edges of the hexagon in this particle’s map counters. $\alpha_{\tilde{h}}^{\tilde{e}}$ and $\alpha_{\tilde{h}}=\sum_{e=0}^{5}\alpha_{\tilde{h}}^{\tilde{e}}$ are the prior counts. The result is used in the particle weight update:

(6) $$w_{k}^{i}\propto w_{k-1}^{i}\cdot I^{i}$$

where $w_{k}^{i}$ denotes the weight of the *i*-th particle at step *k*. If a particle crossed an edge that has been crossed more frequently than the other edges of the previous hexagon, it tends to have more weight. Thus, a consistent walking pattern would be generated.

Each particle contains information about the previous track and the probability of transitions from each hexagon to its adjacent hexagons, which is represented by a probabilistic map. The final result is the best map based on the particle with the highest weight.

## 3. Experimental Conditions

### 3.1. Data Collection Site

The data collection was conducted in two environments, consisting of an indoor and outdoor setting. Indoor tests took place in a building with a known floor plan, whilst the outdoor experiments were conducted using a basketball court, in order to have an exact measurement for the reference map. These maps were used as ground truths to allow for a direct comparison with the PDR and HeadSLAM outcomes.

### 3.2. Participants

There were 5 volunteers for the indoor data collection session and 5 volunteers for the outdoor data session with 2 people participating in both. The demographic information of the participants is given in Table 1. All participants signed a consent form before the data collection started, and they were given the opportunity to ask any questions before deciding to be involved in this study. Ethical approval was obtained from the University Ethics Committee, and this experiment was part of a larger study (R70833/RE001).

### 3.3. Devices

**Figure 2:**
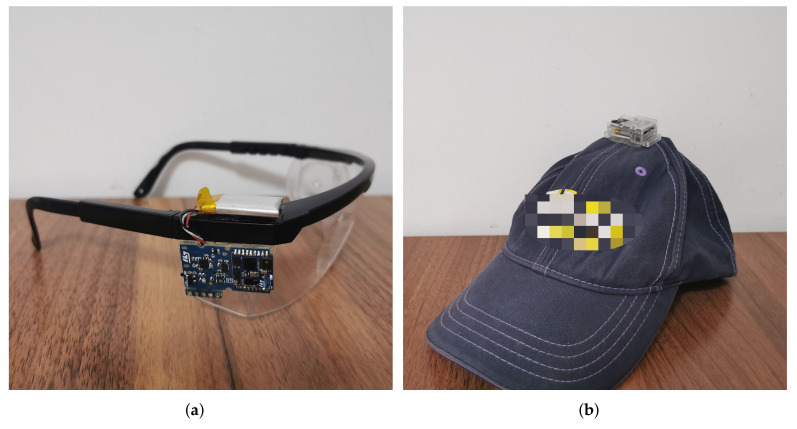
Placement of sensor modules (**a**) on the glasses (**b**) on the cap.

**Figure 3:**
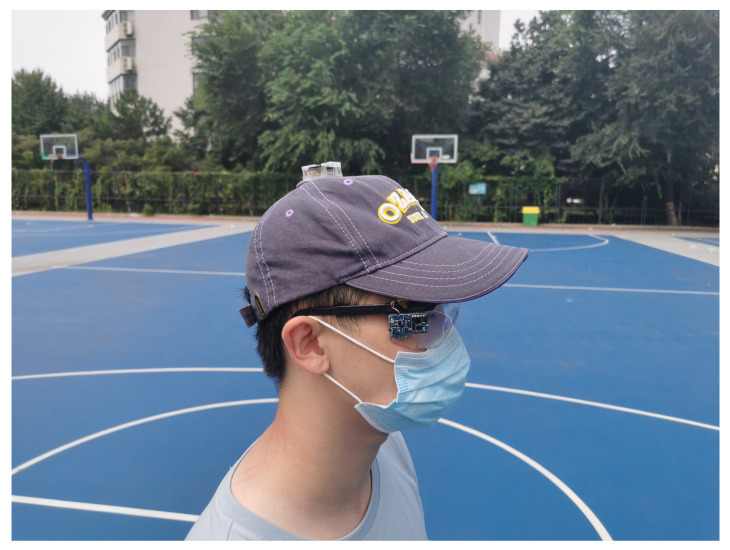
A subject wearing the devices.

The sensor adopted in the experiments was the SensorTile Microcontroller Unit (MCU) module (STEVAL-STLCS01V1) from STMicroelectronics (Geneva, Switzerland), which includes a low-power 3D accelerometer and 3D gyroscope (LSM6DSM), an ultra-low-power 3D magnetometer (LSM303AGR), a Bluetooth low energy network processor (BlueNRG-MS), a 32 bit ultra-low-power MCU with Cortex^®^M4F (STM32L476JG), and a 100 mAh lithium-ion polymer battery. Data were collected at 20 Hz and transferred to a mobile phone by Bluetooth. Two modules were used for each sessions with different placements. One was firmly attached to a pair of glasses, whilst the other was connected to a cap. The placement is shown in Figure 2 and Figure 3.

### 3.4. Experimental Setup

Before the experiments, participants were asked to put on the cap and wear the glasses with the sensors on them. They were requested to place these on their head in such a way that they remained comfortably in contact with the head during walking. Each volunteer was asked to complete the test, for a given environment, six times. Each test took around 10 min. In the first three tests, subjects were requested to walk the same predetermined trajectories. The last three tests consisted of volunteers walking randomly in the indoor space or randomly on the lines of the basketball court (outdoor). In all the tests, participants were instructed to walk at a normal and constant speed whilst keeping their head facing the walking direction. A total of 60 datasets were collected across all experiments. Each dataset contained both the data from the instrumented glasses, as well as from the instrumented cap.

### 3.5. Statistical Analysis

A total of 3 error measurements were computed by comparing the positional outcomes from the algorithms with the known reference map or path. These error measurements consisted of the Root-Mean-Squared Error (RMSE), average absolute error, and max absolute error. All errors were computed across the whole trajectory and in meters. A Kolmogorov–Smirnov test was applied to determine if the data were normally distributed. The Kolmogorov–Smirnov test showed they were not normally distributed, which was confirmed further by visual inspection of the histograms. The errors obtained by the PDR were therefore compared with those of the HeadSLAM using the Wilcoxon signed-rank test. A *p*-value of less than 0.05 was considered significant. All data analysis was conducted in MATLAB (R2020a, Mathworks, Natick, MA, USA).

## 4. Results

**Figure 4:**
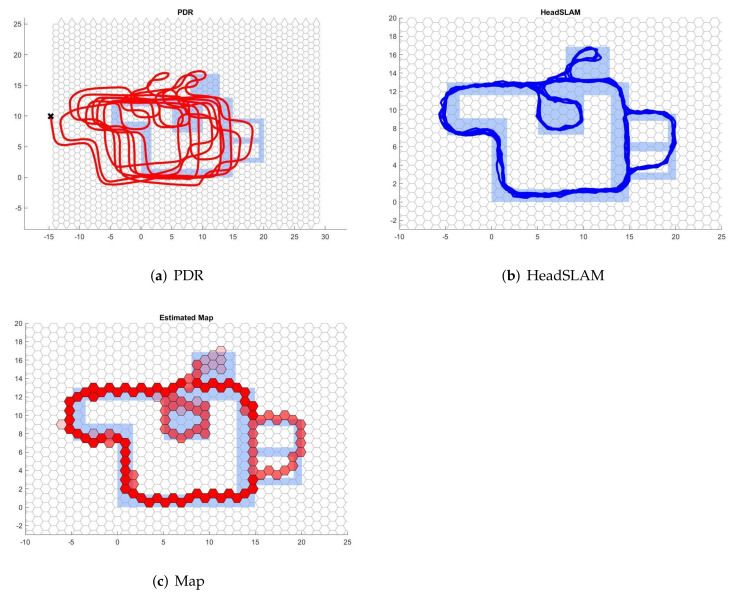
Indoor test results’ example. The light blue shape is the passable area extracted from the floor plan and acts as a reference for the outcomes generated by the PDR and HeadSLAM. Red lines in (**a**) represent the trajectory estimated by the PDR method. Blue lines in (**b**) show the trajectory of the particle with the highest weight in SLAM. Red hexagons in (**c**) represent the trajectory map generated by HeadSLAM, with darker shades representing those with a higher visiting frequency.

**Figure 5:**
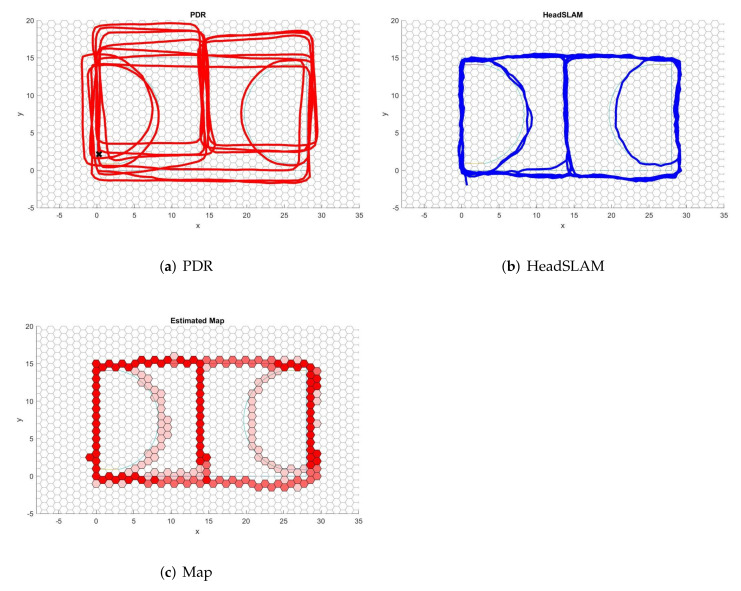
Outdoor test results’ example. Participants were asked to walk the outline of a basketball court. Red lines in (**a**) represent the trajectory estimated by the PDR method. Blue lines in (**b**) show the trajectory of the particle with the highest weight in SLAM. Red hexagons in (**c**) represent the trajectory map generated by HeadSLAM, with darker shades representing those with a higher visiting frequency.

An example of the indoor test results is shown in Figure 4, and for the outdoor test results, an example is shown in Figure 5. The results for all subjects across all tests can be found in Supplementary Materials.

The obtained errors are shown in Table 2 for the two different environments. The ground truths consisted of the floor plan for the indoor environment and the lines on the basketball court for the outdoor environment. The data were split further between the PDR and HeadSLAM methods. The Kolmogorov–Smirnov test showed that a significant difference was present between the PDR and HeadSLAM methods (across all three error measurements) for both the indoor and outdoor environment.

## 5. Discussion

The results showed that HeadSLAM performed better then the PDR across all volunteers and environments. This increase in performance was likely due to HeadSLAM’s efficient calibration approach. However, the effectiveness of HeadSLAM only existed when trajectories on a confined path were repeated and overlapped, such as walking in the corridor for several laps. The overlaps allowed for re-calibration and provided updates to the probability map that was being generated. HeadSLAM is thus applicable for scenarios in which people cover the same path multiple times.

Our dataset, containing around 20 h of walking data (2 devices · 5 subjects · 2 scenarios · 6 repeats · 10 min walking), was much larger than previous studies. Reference [23] used one subject to test indoors and outdoors once with 17 min of walking in total. Reference [22] used one subject to test three times with 30 min of walking in total. A larger dataset is essential to validate the robustness of these systems and provides a better way to ensure they perform across different movement behaviours.

It should be noted that the current approach still requires parameter optimisation. This is currently not conducted in an automated manner. Automatically tuning the parameter is one of the key issues that needs to be solved before a practical application can be considered. Since there are internal drift errors in low-cost sensors and because of external factors that influence the sensors such as temperature, the optimal parameters in Mahony algorithms and the particle filter will be different in each test. FootSLAM could solve this problem by calculating them based on the data collected at the beginning when the foot is kept still. For example, Reference [33] used an adaptive threshold in the ZUPT. However, it is impossible to leverage this technique in HeadSLAM, because the head cannot be kept completely still when the participant is in the standing pose. Fixing the head during a calibration period does not provide a minimally obtrusive method of tracking. This is something that should be explored in further research.

It should also be noted that other parameters also vary between people. The *k* in the step length estimation will differ between individuals. In this study, these parameters were adjusted manually. If a plug-and-play system is required for a better user experience, then all these parameters should be set automatically. Itzik Klein and Omri Asraf proposed a method that uses deep learning to estimate the Weinberg gain [34]. This could be an interesting way of solving some of these issues.

The HeadSLAM approach presented here can be useful for human tracking at scale. It only requires low-cost IMUs, whilst other infrastructure solutions for positional tracking can be expensive to set up and maintain. It is also not prone to privacy issues, which could arise when cameras are used. More importantly, the system can be fully self-contained, creating possibilities for very secure tracking. Although HeadSLAM could work without external infrastructures or previously known information, there is also a possibility to combine it with other methods to create a more accurate and robust system. Switching between HeadSLAM and approaches that require infrastructure can also solve the loss of position estimation whenever, for example, there is a temporarily weak or a loss of the WiFi/GPS signal.

## 6. Conclusions

HeadSLAM could reach an average RMSE of 0.34 m indoors and 0.83 m outdoors during a 10 min walk, which shows a significant improvement compared to the PDR method. It shows the potential of longer-term location services based on head-mounted low-cost sensors, which allow for possible inexpensive applications in healthcare, sports, or emergency services. The particle filter in the HeadSLAM approach smooths out small errors that are due to head motions. However, further research will need to conducted to deal with the problem of unexpected (larger) head movements during walking. These kinds of unique problems need to be addressed to generate real-world impact for head-mounted sensors.

## Figures and Tables

**Table 1:** Demographics of the participants.

Subjects	1	2	3	4	5	6	7
Age	25	20	23	24	49	46	47
Height (m)	1.80	1.81	1.77	1.66	1.60	1.75	1.60
Weight (kg)	80	74	61	58	55	89	62
Gender	M	M	F	F	F	M	F
Indoor tests	✓	✓	✓		✓		✓
Outdoor tests		✓	✓	✓		✓	✓

**Table 2:** Errors in meters (m) are given for the indoor and outdoor environments for both the PDR and HeadSLAM methods. RMSE is the Root-Mean-Squared Error. A significant difference based on the Wilcoxon signed-rank test (*p*-value $1.63\cdot 10^{-11}$) was found for all six direct comparisons between the PDR and HeadSLAM outcomes.

Environment	Algorithm	RMSE	Average Absolute Error	Max Absolute Error
Indoor	PDR	2.2943	1.4108	8.2473
	HeadSLAM	0.3399	0.1610	1.6597
Outdoor	PDR	2.4358	1.7712	7.6218
	HeadSLAM	0.8343	0.6400	2.8368

## Data Availability

The data presented in this study are available on request from the corresponding author. The data are not publicly available due to privacy.

## Supplementary Materials

The following are available online at https://doi.org/10.5281/zenodo.5562364, Results of all datasets.
